# Genetic Background of Acute Heart Rate Response to Exercise

**DOI:** 10.3390/ijms25063238

**Published:** 2024-03-13

**Authors:** Péter Pikó, Habib Al Ashkar, Nóra Kovács, Ilona Veres-Balajti, Róza Ádány

**Affiliations:** 1 Department of Public Health and Epidemiology, Faculty of Medicine, University of Debrecen, 4032 Debrecen, Hungary; kovacs.nora@med.unideb.hu (N.K.); adany.roza@med.unideb.hu (R.Á.); 2 National Laboratory for Health Security, Center for Epidemiology and Surveillance, Semmelweis University, 1089 Budapest, Hungary; 3 HUN-REN-UD Public Health Research Group, Department of Public Health and Epidemiology, Faculty of Medicine, University of Debrecen, 4032 Debrecen, Hungary; axarhabib@gmail.com; 4 Department of Physiotherapy, Faculty of Health Sciences, Institute of Health Sciences, University of Debrecen, 4028 Debrecen, Hungary; balajti.ilona@etk.unideb.hu; 5 Department of Public Health, Semmelweis University, 1089 Budapest, Hungary

**Keywords:** acute heart rate response, cardiorespiratory fitness, YMCA 3 min step test, optimised polygenic score, single nucleotide polymorphism, leisure-time physical activity

## Abstract

The acute heart rate response (AHRR) to physical activity, which refers to the change in heart rate during and after exercise, has been associated with cardiovascular and all-cause mortality. Previous studies have shown that AHRR is significantly determined by genetics in addition to environmental and lifestyle factors. The aim of this study was to investigate the genetic background of AHRR by analysing ten single nucleotide polymorphisms (SNPs) associated with leisure-time physical activity (LTPA) in 620 samples from the Hungarian population. The AHRR can be characterised as the difference between post-exercise and resting heart rate, i.e., the delta heart rate (ΔHR) defined by the YMCA 3 min step test, with a lower value indicating better cardiovascular fitness. The association of SNPs with ΔHR was analysed both separately and in combination using an optimised polygenic score (oPGS). The results showed that five SNPs (rs10252228, rs459465, rs6022999, rs8097348, and rs12405556) had at least nominally significant (*p* < 0.05) individual associations with ΔHR. After optimizing the PGS, a cumulative effect was observed for eight SNPs (rs6022999, rs12405556, rs459465, rs10252228, rs8097348, rs10887741, rs12612420, and rs7023003) that had a strong and statistically significant association with ΔHR (B = −2.51, 95% CI: −3.46–−1.76; *p* = 2.99 × 10^−9^). Of the four main domains of physical activity, the oPGS showed a significant positive association only with LTPA (B = 84.60; 95%CI: 25.23–143.98; *p* = 0.005). In conclusion, our results suggest that the SNPs we investigated influence individual leisure-time physical activity, mediated by their effects on the acute heart rate response.

## 1. Introduction

In recent years, adverse effects of urbanisation and technological innovations [1], as well as restrictions during the COVID-19 pandemic [2], have contributed to a radical decline in physical activity. As a result, insufficient physical activity has become one of the biggest global public health challenges of our time [3].

It is well known that physically active individuals have a significantly lower risk of morbidity and mortality from cardiovascular diseases compared to their sedentary counterparts. This cardioprotective effect is usually attributed to improvements in traditional risk factors for cardiovascular diseases [4], and regular physical activity has a positive impact on heart functions. Physically active individuals can perform the same physical work with less cardiac workload, as evidenced by a lower heart rate and blood pressure for a given workload compared to sedentary individuals. Animal studies have demonstrated that a decreasing heart rate can have direct health benefits. For instance, in cynomolgus monkeys, reducing the heart rate through sinoatrial node ablation slowed down and even prevented atherosclerotic lesions induced by a high-fat diet in coronary arteries [5] and carotid bifurcation [6]. In human studies, it was also convincingly demonstrated that even after adjustment for the most important cardiovascular risk factors, increased heart rate remained an independent predictor of adverse events in the global population or patients with cardio- and cerebrovascular diseases [7].

The regulation of heart rate is a complex process influenced by a combination of different factors (hormonal effects, central and peripheral reflexes, and autonomic tone) [8]. The acute heart rate response (AHRR), characterised by the difference between the heart rate measured immediately before and after a standardised form of exercise (ΔHR) [9], has been linked to morbidity caused by different—mainly cardiovascular—diseases and to all-cause mortality [10,11,12]. Regular aerobic exercise has been shown to shift the autonomic balance of the heart towards vagal dominance [13]. The extended reaction to physical activity yields advantageous modifications in chronotropic function, leading to a decrease in resting and sub-maximal heart rate, along with an enhanced recovery [4].

The positive association between physical activity and AHRR has long been known [14,15]. However, different forms of physical activity are not equally associated with cardiovascular protection. There are two main approaches to studying the protective effects of physical activity. The first examines the effect in relation to the domains of physical activity, namely work, travel, household and garden, and leisure-time physical activity (LTPA), while the second examines the effect in terms of intensity categories, namely vigorous, moderate, and light. Of the domains, domestic/gardening and LTPA were found to be protective [16,17,18,19], while transport and work were found to be marginally or not at all [20,21,22]. Of the intensity categories, moderate and vigorous showed significant associations with reduced cardiovascular risk and mortality [23,24,25].

Both the immediate and prolonged heart rate response to physical activity have been demonstrated to have a hereditary component [26,27,28,29] in addition to environmental and lifestyle factors. According to a 2019 review [26], a total of ten genes in candidate gene studies were associated with AHRR, while a further 17 candidate causative genes in genome-wide association studies (GWAS) were identified as being associated with heart rate elevation and 26 as being associated with heart rate recovery. A further ten genes were associated with the long-term modification of the heart rate response to exercise, nine with an increase in the heart rate, and one with the recovery of the heart rate.

Among the domains of physical activity, LTPA is the one that can be tested freely by the individual without external influences (physical work for a living, travel for work, etc.); thus, it has become a major target of genetic research. LTPA is influenced by a combination of lifestyle and environmental factors, but studies estimate its genetic heritability to be between 30% and 52% [30]. There are several polymorphisms known to be closely associated with individual LTPA levels [31,32,33,34]. A common feature of the above studies is that they identify polymorphisms associated with LTPA but do not provide a sufficient explanation of their mechanism of action.

The cardioprotective effect of LTPA, the fact that AHRR is strongly associated with cardiovascular fitness, and the fact that both LTPA and AHRR are genetically determined raise the question: are genetic factors associated with LTPA directly related to individual AHRR?

The present study is the first to investigate whether there is an association between AHRR and selected SNPs that showed a strong association with LTPA and to confirm our hypothesis that these polymorphisms exert their beneficial effect by positively influencing AHRR.

## 2. Results

### 2.1. Characteristics of the Study Populations

Samples with incomplete genotype and/or phenotype data were excluded from further analyses; thus, a total of 620 individuals were involved in the present study.

There was no significant difference (after Bonferroni correction: *p* < 0.00625) in basic characteristics between the three groups defined as people with poor, moderate, and good AHRR (see Table 1 for details).

Except for resting heart rate, all heart rate parameters characterising cardiovascular fitness showed a significant trend change between groups (see Table 2 for details).

For the AHRR subgroups, a significant positive trend was measured for the total physical activity (*p* = 2.00 × 10^−5^), vigorous (*p* = 5.19 × 10^−4^) and moderate intensity (*p* = 4.00 × 10^−6^) categories, as well as for work (*p* = 0.0025) and leisure-time physical activity (*p* = 8.14 × 10^−4^). See Table 3 for details.

### 2.2. Result of Hardy–Weinberg Analysis and Individual Association of SNPs with AHRR and Optimisation of Polygenic Score

No significant (*p* < 0.00625) deviation from the Hardy–Weinberg equilibrium (HWE) was found for the ten SNPs tested in terms of genotype distribution. Five showed at least a nominally significant (*p* < 0.05) association with ΔHR, but after adjustment for test correction (*p* < 0.00625), only rs6022999 showed a significant (B = −6.365, 95% CI: −9.369–−3.361; *p* = 3.60 × 10^−5^) association. See Supplementary Table S1 for more details.

In the optimisationoptimisation of the PGS, we sought to select SNPs that, based on linear regression analysis, strengthen the association between oPGS and ΔHR. Starting from the SNP with the strongest association (rs6022999) to the weakest one (rs7023003), we added each SNP one by one into the statistical model (adjusted for ethnicity, sex, age, travelling by vehicle, total physical activity in metabolic equivalent task minutes per week (MET-min/week), body mass index (BMI), education, diastolic blood pressure, fasting glucose, and current smoking status). All SNPs that increased the association of the model (decreased *p*-value and increased R-square) were retained and used to calculate the oPGS. Conversely, those SNPs that decreased the association of the model (increased *p*-value and decreased R-square) were excluded. During the optimisation process, eight SNPs were selected (rs6022999, rs12405556, rs459465, rs10252228, rs8097348, rs10887741, rs12612420, and rs7023003). Compared to the baseline reference SNP (rs6022999), oPGS shows a stronger significant association with ΔHR (B = −2.61, 95%CI: −3.46–−1.75; *p* = 3.29 × 10^−9^), with improved predictive value (R^2^_rs6022999_: 0.084 vs. R^2^_oPGS_: 0.110). For more details, see Supplementary Table S2.

The oPGS (both as continuous and categorical variables) showed a significant correlation with all heart rate indices except resting pulse. The oPGS showed the strongest correlation with heart rate measured immediately after physical exercise (B = −2.863, −3.730–−1.996; *p* = 1.83 × 10^−10^). See Table 4 and Supplementary Table S3 for more details.

### 2.3. Association of oPGS with the Categories and Domains of Physical Activity

The oPGS showed no significant association with total physical activity and its intensity-based subcategories. Of the four main domains (work, transport, housework/gardening, and leisure-time physical activity), the oPGS showed a significant association only with LTPA (B = 84.85, 95% CI: 25.43–144.27; *p* = 0.005). See Table 5 for more details.

After adjusting the statistical model for ΔHR (as a possible independent influencing factor), oPGS was no longer significantly correlated with LTPA (B = 50.25; 95%CI: −9.99–110.49; *p* = 0.102).

Based on the results of the trend analysis, a significant association was found between oPGS subgroups and physical activity in MET-min/week only for the vigorous intensity category and LTPA. See Supplementary Table S4 for details.

Among the intensity categories, only vigorous showed a significant (*p* = 0.006) trend correlation with oPGS groups, while in the case of domains, only LTPA showed a significant (*p* = 5.11 × 10^−4^) trend correlation. See Supplementary Table S5 for details.

### 2.4. The association of oPGS with AHRR, Independent of Individual Physical Activity

The association of the oPGS with AHRR, independent of physical activity, was examined in three statistical models. The oPGS showed a significant association with AHRR in all models. Independent of oPGS, total physical activity, vigorous and moderate intensity categories, and LTPA showed significant associations with AHRR. See Table 6. for more details.

## 3. Discussion

Physical activity, especially recreational activity, has a positive effect on cardiovascular fitness and thus reduces the risk of developing cardiovascular disease. Individual LTPA levels are partly genetically determined. The present study aimed to investigate the effect of ten SNPs strongly associated with LTPA on AHRR.

Only one of the ten SNPs examined, rs6022999 in the *CYP24A1* gene, showed an individually significant association with AHRR. The protein transcribed from the *CYP24A1* gene is an enzyme expressed in the mitochondria that catalyses hydroxylation reactions leading to the breakdown of the physiologically active form of vitamin D, 1,25-dihydroxyvitamin D3. Side-chain hydroxylation results in calcitric acid and other metabolites, which are excreted in the bile. In addition to the effect of rs6022999 on LTPA [35], it has also been associated with an increased risk of liver [36], lung [36] and colorectal [37] cancers and hepatitis C infection.

During the optimisation of the polygenic risk score, we identified eight SNPs whose combined effect showed a significant association with AHRR. The oPGS showed the strongest association with heart rate measured after immediate physical activity. The oPGS showed a significant association with LTPA, but this association disappeared after correction for ΔHR. This suggests that the significant association of oPGS with LTPA is not independent of its effect on delta heart rate.

The results of our analysis show that oPGS significantly affects AHRR independent of physical activity intensity categories or domains. Total physical activity had a significant and positive effect on AHRR. When intensity categories were examined, vigorous and moderate-intensity exercise had a positive effect on AHRR, which is supported by previous research [38]. All four main physical activity domains were positively associated with AHRR (reduced delta HR), but this was only significant for LTPA.

Research into the genetic basis of leisure-time physical activity goes back several decades. Candidate gene and GWAS studies have identified several gene polymorphisms associated with LTPA. A common feature of these studies is that the effects of the identified genomic elements on LTPA are difficult or impossible to explain by direct correlation with biological pathways. Possible explanatory pathways could include direct or indirect associations with the reward system [39], energy balance [30], bone [40], and muscle development [30]. Our results may partially explain the underlying processes, as they show that the SNPs associated with LTPA that we investigated are related to AHRR and, thus, to cardiovascular fitness.

This study has both strengths and limitations. First, due to the lack of information on gene-gene and gene-environment interactions, epigenetic factors, and structural variants, these were not considered in our analysis. In the current study, we included ten SNPs related to LTPA in the calculation of oPGS. The inclusion of a larger number of SNPs may further improve the predictive ability of the PGS model. However, adding more SNPs to the PGS model does not necessarily lead to better predictive ability, as shown during the optimisation process. Another limitation is that individuals over 65 years of age were not included in our present study. In addition, as a limitation, it must be acknowledged that since the sample included only 215 men, this number is not sufficient to draw firm conclusions about separate sexes. Despite the limitations of this study, it should be emphasised that this is the first study to investigate the association of AHRR and LTPA with genetic factors. Furthermore, it tests the hypothesis that polymorphisms closely associated with LTPA may influence an individual’s physical activity through their beneficial effects on heart rate variability.

In conclusion, we have successfully demonstrated that genetic factors can significantly influence individual heart rate response/variability independent of the intensity and type of physical activity. Furthermore, it has been demonstrated that the effect of LTPA-promoting polymorphisms is partly mediated through their beneficial effects on heart rate variability. However, further independent studies are needed to confirm these findings.

## 4. Materials and Methods

### 4.1. Sample Population and Investigations Performed

The data for our study were obtained in a 2018 complex (health examination and health behaviour) cross-sectional survey, which consisted of a three-pillar approach (questionnaire, physical examination, and laboratory examination). Further details on the sampling and data collection process are provided elsewhere [41].

Briefly, the study recruited samples from two counties (Hajdú-Bihar and Szabolcs-Szatmár-Bereg) in northeastern Hungary, which have the highest Roma representation and segregated Roma colonies. The Hungarian general (HG) population sample consisted of randomly selected individuals aged between 20 and 64, living in private households, and registered with general practitioners. For the study, 25 randomly selected individuals were invited to participate in each of the 20 randomly selected GP practices. In addition, 25 colonies were also randomly selected, and one person (20–64 years of age) from each of the 20 households (also randomly selected) per colony was invited to participate in the survey. The ethnicity of the participants was self-reported. The target sample size for the survey was 500 individuals per population. However, the final study sample size was reduced to 797, consisting of 410 participants from the HG population and 387 participants from the Roma population, due to the exclusion of individuals with incomplete records.

The primary instrument used in the complex survey was the questionnaire for the European Health Interview Survey Wave 2 (EHIS 2), featuring four modules:

(a) health status;
(b) health care utilisation;
(c) health determinants;
(d) socioeconomic measures.

The blood samples collected were analysed for routine laboratory parameters (described in detail in [41]) including fasting blood glucose, and used for DNA extraction.

### 4.2. Measurement of Physical Activity and Cardiovascular Fitness

The EHIS 2 questionnaire was extended with additional question sets, including the comprehensive edition of the International Physical Activity Questionnaire (IPAQ) to assess physical activity across domains and dimensions [42].

The IPAQ questionnaire is designed to assess the time spent engaging in light, moderate-intensity, and vigorous-intensity activities in the past week across different domains (work, transportation, domestic and garden tasks, leisure, and time spent sitting). The IPAQ data were processed using the standardised IPAQ Scoring Protocol. Only activities lasting more than ten minutes during the previous seven days were recorded in the questionnaire. The results of the questionnaire were used to calculate MET-min/week [43,44].

The YMCA 3 min step test was used to measure cardiovascular fitness. This type of examination measures submaximal cardiorespiratory or endurance fitness.

The test steps were:

(1) Each test starts with a 2 min rest period while the subjects sit on a chair.
(2) Subjects are required to step up and down a 30 cm box 72 times in 3 min. The step rate was indicated by a metronome set at 96 beats/min (4 clicks = one step cycle) at a step rate of 24 steps/min.
(3) The subject stops immediately after the test is completed, sits down and remains motionless for 5 s, and then the subject’s pulse is monitored for one minute.
(4) The heart rate measurement is repeated 5 and 10 min later.

The AHRR was assessed as the difference between post-exercise (5 sec) and resting (5 min, 10 min) heart rate, which was expressed as delta heart rate (ΔHR). ΔHR shows an inverse association with cardiovascular fitness [45].

Individuals in the study populations were ranked by ΔHR (from higher to lower) and divided into three AHRR-related categories: poor, moderate, and good. These groups were compared, and trend analysis was used to examine differences in factors relevant to the aim of the study.

### 4.3. DNA Extraction, SNP Selection, Genotyping, Testing Hardy–Weinberg Equilibrium, and Linkage Disequilibrium

The MagNA Pure LC system (Roche Diagnostics, Basel, Switzerland) was used to extract DNA from EDTA-anticoagulated blood samples according to the manufacturer’s instructions.

SNPs significantly associated with LTPA were identified through a systematic literature search using online search engines including PubMed, Ensemble, and HuGE Navigator. The search for this study was conducted until 5 August 2019. The search terms used in this study were ‘leisure-time physical activity’, ‘recreational physical activity’, ‘genetics’, ‘GWAS’, ‘candidate gene’, and ‘genotype’. Special attention was given to selecting SNPs based on the results of three GWAS [32,33,34] and one candidate gene study [35], which were the most relevant in this area.

Ten SNPs were genotyped at the Mutation Analysis Core Facility (MAF) at Karolinska University Hospital, Sweden, using the MassARRAY platform (Sequenom Inc., San Diego, CA, USA) and iPLEX Gold chemistry. The MAF conducted validation, concordance analysis, and quality control following their protocols. The genotyped SNPs were analysed for HWE structure and linkage disequilibrium (LD) using Haploview software (version 4.2; Broad Institute; Cambridge, MA, USA).

### 4.4. Calculation and Optimisation of the Polygenic Score

Participants with any missing SNP genotypes were excluded from subsequent analyses, leaving a total of 317 individuals from the HG sample and 303 Roma participants for the genotype analysis. During the PGS calculations, scores were assigned to each person based on the number of effect alleles they carried. The allele promoting AHRR was considered to be the effect allele.

Each SNP has been coded according to the criteria of the genetic model of inheritance. Therefore, for the

- Codominant genetic model: homozygous genotype with risk allele was coded as 2, while the heterozygous gene was coded as 1 and 0 was coded for no risk allele.
- Dominant genetic model: 2 was coded for the presence of one or two risk alleles, and 0 was coded for the absence of a risk allele.
- Recessive genetic model: 2 was scored for the presence of two risk alleles, while 0 was scored for the homozygous gene with no risk allele and the heterozygous gene.

Using these codes, a simple count score was calculated as described in Equation (1), where Gi is the number of effect alleles for the ith SNP. This model sums all alleles across all loci as a summary score, assuming that all alleles have the same effect in direction and magnitude.

*GRS* = ∑*Ii* = 1*Gi* (1)

The polygenic model optimisation procedure aimed to select SNPs (identified by the systematic literature search) that had a strong association with AHRR in both study populations. For PGS optimisation, adjusted linear regression analyses (for ethnicity, sex, age, travel by vehicle, total physical activity in MET-min/week, BMI, education, diastolic blood pressure, fasting glucose, and current smoking status) were used, and these analyses were also performed on a combined sample of the two sample populations.

The SNPs were tested in ascending order of *p*-values. Each SNP was inserted into the statistical model one at a time, starting with the SNP with the strongest association (with the lowest *p*-value). The association between oPGS and LTPA was examined after each insertion.

SNPs were selected and used in the final oPGS only if they increased the strength of the association of oPGS (decreased *p*-value and increased Cox-Snell R-square) with AHRR. SNPs that did not weaken the model association, i.e., those that resulted in an increased *p*-value and decreased R-squared, were excluded from further analyses.

Genetic predisposition categories were formed based on the population distribution of oPGS. Four groups were created, and trend analysis was used to examine the association of these groups with AHRR in general and its intensity categories.

### 4.5. Statistical Analysis

The study utilised the χ^2^ test to compare non-quantitative variables and examine the HWE of genotyped SNPs. The Online Sample Size Estimator (OSSE) tool (http://osse.bii.a-star.edu.sg/calculation1.php, accessed on 10 January 2023) was used to calculate statistical power for each SNP. Additionally, the study employed the Shapiro–Wilk test to assess the normal distribution of quantitative variables. Templeton’s two-step method [46] was used to transform non-normal variables into normal variables where necessary.

Multiple linear regression analyses were performed to investigate the association between genetic factors (individual SNPs and oPGS) and AHRR. The regression analyses were adjusted for relevant factors, including ethnicity, sex, age, transport, total physical activity in MET-min/week, BMI, education, diastolic blood pressure, fasting glucose, and current smoking status. The Jonckheere-Terpstra trend test [47] was used to analyse the association trend between oPGS categories and MET-min/week values. The text adheres to conventional structure, clear and objective language, formal register, and precise word choice. The grammar, spelling, and punctuation are correct. Ethnicity was used as a covariate when the two populations were combined and examined together. No changes in content were made. Statistical analyses were conducted using IBM Statistical Package for the Social Sciences (SPSS) version 26 (Armonk, NY, USA). For multiple statistical analyses involving the oPGS, the Bonferroni correction method was used. This required dividing the conventional *p*-value of 0.05 by the number of independent polymorphisms.

## Figures and Tables

**Table 1:** Basic characteristics of the three groups based on acute heart rate response (AHRR—assessed as the difference between post-exercise and resting heart rate—delta heart rate—ΔHR).

		Poor AHRR (n = 206)	Moderate AHRR (n = 208)	Good AHRR (n = 206)	
			Average (95%CI)		*p* for Trend
ΔHR ^¥^		61.42 (58.17–64.66)	26.54 (25.98–27.11)	12.72 (12.07–13.37)	<0.001 **
Age (years)		42.72 (40.96–44.48)	43.88 (42.22–45.53)	42.55 (40.85–44.26)	0.938
Body mass index (kg/m^2^)		27.49 (26.60–28.37)	27.44 (26.70–28.18)	26.89 (26.09–27.70)	0.384
Average diastolic blood pressure (mmHg)		80.21 (78.95–81.47)	78.78 (77.61–79.95)	79.11 (77.69–80.53)	0.321
Fasting glucose (mmol/L)		5.08 (4.88–5.28)	5.17 (4.89–5.45)	5.10 (4.89–5.30)	0.757
			Prevalence in % (95%CI)		*p* for Trend
Women		71.36 (64.92–77.20)	63.94 (57.26–70.24)	60.68 (53.90–67.16)	0.023 *
Traveling by vehicle		44.17 (37.51–51.00)	59.13 (52.37–65.65)	47.09 (40.35–53.90)	0.555
Education	Primary	56.31 (49.49–62.96)	38.46 (32.05–45.20)	64.08 (57.37–70.40)	0.041 *
	High school	30.58 (24.59–37.11)	51.92 (45.15–58.65)	29.13 (23.24–35.59)	
	University	13.11 (9.02–18.22)	9.62 (6.17–14.18)	6.80 (3.95–10.85)	
Current smoking status		47.09 (40.35–53.90)	39.61 (33.13–46.38)	57.28 (50.46–63.90)	0.039 *
Roma ethnicity		53.88 (47.06–60.60)	31.25 (25.24–37.77)	61.65 (54.89–68.09)	0.115

¥: the parameter used to create the subgroups; 95%CI: 95% confidence interval; *: *p* < 0.05; **: significant result after Bonferroni adjusted *p*-value (<0.00625).

**Table 2:** Results of trend comparison of heart rate values measured in YMCA 3 min step test for acute heart rate response (AHRR) subgroups.

	Poor AHRR (n = 206)	Moderate AHRR (n = 208)	Good AHRR (n = 206)	*p* for Trend
	Average (95%CI)			
HR_rest_	76.79 (75.47–78.10)	76.99 (75.54–78.43)	78.56 (77.13–80.00)	0.085
HR_exerc_	138.20 (134.82–141.58)	103.53 (101.99–105.07)	91.21 (89.61–92.80)	<0.001 **
HR_5min_	105.68 (103.08–108.28)	89.44 (88.02–90.85)	84.40 (82.89–85.90)	<0.001 **
HR_10min_	87.18 (85.34–89.02)	80.47 (79.14–81.80)	78.52 (77.13–79.91)	<0.001 **
ΔHR_5min_	28.90 (26.38–31.41)	12.45 (11.36–13.54)	5.83 (5.04–6.63)	<0.001 **
ΔHR_10min_	10.39 (8.87–11.92)	3.49 (2.62–4.35)	−0.04 (−0.74–0.65)	<0.001 **

HR_rest_: resting heart rate; HR_exerc_: heart rate immediately after completing the physical exercise; HR_5min_: heart rate 5 min after the physical exercise; HR_10min_: heart rate 10 min after the physical exercise; ΔHR: delta heart rate, defined as the difference between the heart rate immediately after completing the physical exercise and the resting heart rate; ΔHR_5min_: defined as the difference between the heart rate 5 min after physical exercise and the resting heart rate; ΔHR_10min_: defined as the difference between the heart rate 10 min after physical exercise and the resting heart rate. 95%CI: 95% confidence interval; **: significant *p*-value (<0.00625) after Bonferroni correction.

**Table 3:** Results of trend comparison of total physical activity, intensity, and domain categories for acute heart rate response (AHRR) subgroups.

	Poor AHRR (n = 206)	Moderate AHRR (n = 208)	Good AHRR (n = 206)	
	Average MET-min/week (95%CI)			*p* for trend
Total physical activity	8489 (7540–9439)	11,446 (10,328–12,565)	11,747 (10,655–12,840)	<0.001 **
By intensity categories	Average MET-min/week (95%CI)			*p* for trend
Vigorous	2276 (1592–2960)	3448 (2788–4109	3478 (2739–4218)	<0.001 **
Moderate	4395 (3847–4943)	5811 (5179–6443)	6546 (5848–7245)	<0.001 **
Light	1819 (1517–2120)	2187 (1872–2501)	1723 (1436–2010)	0.944
By domains	Average MET-min/week (95%CI)			*p* for trend
Work	3782 (2984—4580)	5407 (4569–6246)	5510 (4678–6343)	0.003 **
Transport	1283 (1045–1521)	1445 (1180–1710)	1761 (1466–2055)	0.012 *
Domestic work and gardening	2584 (2199–2968)	3241 (2852–3630)	3049 (2628–3470)	0.052
Leisure-time	841 (637–1045)	1353 (1076–1630)	1427 (1167–1688)	<0.001 **

MET-min/week: metabolic equivalent task minutes per week; 95%CI: 95% confidence interval; *: *p* < 0.05; **: significant *p*-value (<0.00625) after Bonferroni correction.

**Table 4:** Association of optimised polygenetic score (oPGS) with heart rate response.

	B Value (95% CI)	*p*-Value
HR_rest_	−0.13 (−0.48–0.22)	0.465
HR_exerc_	−2.86 (−3.73–−2.00)	1.83 × 10^−10^ **
HR_5min_	−1.55 (−2.11–−1.00)	5.15 × 10^−8^ **
HR_10min_	−0.68 (−1.07–−0.28)	0.0008 **
ΔHR	−2.61 (−3.46–−1.75)	3.29 × 10^−9^ **
ΔHR_5min_	−1.41 (−1.95–−0.88)	3.17 × 10^−7^ **
ΔHR_10min_	−0.52 (−0.84–−0.20)	0.001 **

HR_rest_: resting heart rate; HR_exerc_: heart rate immediately after completing the physical exercise; HR_5min_: heart rate 5 min after the physical exercise; HR_10min_: heart rate 10 min after the physical exercise; ΔHR: delta heart rate, defined as the difference between the heart rate immediately after completing the physical exercise and the resting heart rate; ΔHR_5min_: defined as the difference between the heart rate 5 min after physical exercise and the resting heart rate; ΔHR_10min_: defined as the difference between the heart rate 10 min after physical exercise and the resting heart rate. 95%CI: 95% confidence interval; **: significant *p*-value (<0.00625) after Bonferroni correction.

**Table 5:** Association of oPGS with total physical activity, physical activity intensity categories, and domains.

	B value (95%CI)	*p*-value
Total physical activity	179.28 (−95.94–454.50)	0.201
By intensity categories	B value (95%CI)	*p*-value
Vigorous	−51.63 (−197.48–94.21)	0.490
Moderate	58.46 (−92.21–208.92)	0.447
Light	−3.81 (−80.21–72.58)	0.922
By domains	B value (95%CI)	*p*-value
Work	−42.64 (−231.95–146.66)	0.658
Transport	40.90 (−22.68–104.48)	0.207
Domestic and gardening	44.62 (−56.07–145.31)	0.385
Leisure-time	84.85 (25.43–144.27)	0.005 **

95%CI: 95% confidence interval; **: significant *p*-value (<0.00625) after Bonferroni correction.

**Table 6:** Results of the association of oPGS as a genetic factor independent of physical activity with acute heart rate response.

	B value (95%CI)	*p*-value
Total physical activity	−0.001 (−0.001–0.000)	1.90 × 10^−5^ **
oPGS	−2.606 (−3.458–−1.754)	3.29 × 10^−9^ **
By intensity categories	B value (95%CI)	*p*-value
Vigorous	−0.002 (−0.003–−0.001)	5.37 × 10^−4^ **
Moderate	−0.002 (−0.003–−0.001)	0.002 **
Light	−0.002 (−0.004–0.000)	0.085
oPGS	−2.698 (−3.548–−1.849)	8.33 × 10^−10^ **
By domains	B value (95%CI)	*p*-value
Work	−0.000 (−0.001–0.000)	0.109
Transport	−0.000 (−0.002–0.001)	0.394
Domestic work and gardening	−0.001 (−0.001–0.000)	0.066
Leisure-time	−0.002 (−0.003–−0.001)	6.35 × 10^−4^ **
oPGS	−2.498 (−3.350–−1.646)	1.37 × 10^−8^ **

95%CI: 95% confidence interval; **: significant *p*-value (<0.00625) after Bonferroni correction. All statistical models are adjusted for ethnicity, sex, age, travel by vehicle, body mass index, education, diastolic blood pressure, fasting glucose, and current smoking status in addition to those listed in the table.

## Data Availability

Data are available on request due to privacy or ethical concerns.

## Supplementary Materials

The following supporting information can be downloaded at: https://www.mdpi.com/article/10.3390/ijms25063238/s1.
