# Supplementary material for: Genetic Background of Acute Heart Rate Response to Exercise
Source: Int J Mol Sci. 2024 Mar 13;25(6):3238. doi: 10.3390/ijms25063238 (PMC10970476; doi:10.3390/ijms25063238)
Supplement: Supplementary file 1 [file ijms-25-03238-s001.zip › ijms-2901201-supplementary/Supplementary Table S1.docx]

**Supplementary Table S1.** Individual effects of SNPs on acute heart rate response (delta heart rate - ΔHR) based on the result of adjusted (by ethnicity, sex, age, traveling by vehicle, total physical activity in MET-min/week, body mass index, education, diastolic blood pressure, fasting glucose, and current smoking status) linear regression analyses.

| SNP (effect allele) | Gene | Genetic model | B value (95%CI) | *p*-value | R-square |
| --- | --- | --- | --- | --- | --- |
| rs6022999 (A) | *CYP24A1* | Codominant | -6.39 (-9.40 – -3.39) | 3.40 × 10^-5^** | 0.0841 |
| rs12405556 (G) | *LEPR* | Codominant | -4.49 (-7.78 – -1.20) | 0.008* | 0.0690 |
| rs459465 (A) | intergenic | Recessive | -2.73 (-4.83 – -0.63) | 0.011* | 0.0680 |
| rs10252228 (G) | intergenic | Dominant | -2.46 (-4.45 – -0.46) | 0.016* | 0.0670 |
| rs8097348 (G) | *C18orf2* | Dominant | -4.78 (-9.17 – -0.39) | 0.033* | 0.0650 |
| rs6092090 (T) | intergenic | Recessive | -1.59 (-3.56 – 0.38) | 0.114 | 0.0620 |
| rs10887741 (C) | *PAPSS2* | Dominant | -1.66 (-3.66 – 0.34) | 0.113 | 0.0618 |
| rs12612420 (G) | *DNAPTP6* | Recessive | -1.56 (-3.54 – 0.41) | 0.121 | 0.0618 |
| rs429358 (C) | *APOE* | Dominant | -1.35 (-4.26 – 6.96) | 0.637 | 0.0582 |
| rs7023003 (G) | intergenic | Recessive | -0.33 (-5.65 – 4.99) | 0.903 | 0.0579 |

MET-min/week: metabolic equivalent task minutes per week; 95%CI: 95% confidence interval; *: *p* <0.05; **: significant *p*-value (<0.00625) after Bonferroni correction, *CYP24A1*: cytochrome P450 family 24 subfamily A member 1; *LEPR*: Leptin receptor; *PAPSS2:* 3'-Phosphoadenosine 5'-Phosphosulfate Synthase 2; *DNAPTP6:* DNA polymerase transactivated protein 6*; APOE:* Apolipoprotein E*;* intergenic: intergenic region.
