# Supplementary material for: Genetic Background of Acute Heart Rate Response to Exercise
Source: Int J Mol Sci. 2024 Mar 13;25(6):3238. doi: 10.3390/ijms25063238 (PMC10970476; doi:10.3390/ijms25063238)
Supplement: Supplementary file 1 [file ijms-25-03238-s001.zip › ijms-2901201-supplementary/Supplementary Table S2.docx]

**Supplementary Table S2.** List of SNPs (coded by the most fitting genetic model of inheritance) and their effect on the acute heart rate response (delta heart rate - ΔHR) by adjusted (by ethnicity, sex, age, traveling by vehicle, total physical activity in MET-min/week, body mass index, education, diastolic blood pressure, fasting glucose, and current smoking status) linear regression model in order of *p*-value (from the lowest value to the highest one).

| SNP (effect allele) | Genetic Model | B value (95%CI) | *p*-value | R-square | Include/exclude |
| --- | --- | --- | --- | --- | --- |
| rs6022999 (A) | Codominant | -6.37 (-9.37 – -3.36) | 3.60 × 10^-5^ | 0.0841 (ref.) | Included |
| rs12405556 (G) | Codominant | -5.64 (-7.87– -3.42) | 8.07 × 10^-7^ | 0.0951 (↑) | Included |
| rs459465 (A) | Recessive | -3.82 (-5.28 – -2.35) | 4.00 × 10^-7^ | 0.0971 (↑) | Included |
| rs10252228 (G) | Dominant | -3.16 (-4.30 – -2.02) | 7.31 × 10^-8^ | 0.1021 (↑) | Included |
| rs8097348 (G) | Dominant | -3.21 (-4.30 – -2.12) | 1.09 × 10^-8^ | 0.1075 (↑) | Included |
| rs6092090 (T) | Recessive | -2.66 (-3.58 – -1.74) | 2.16 × 10^-8^ | 0.1069 (↓) | Excluded |
| rs10887741 (C) | Dominant | -2.79 (-3.73 – -1.85) | 9.88 × 10^-9^ | 0.1078 (↑) | Included |
| rs12612420 (G) | Recessive | -2.51 (-3.35 – -1.67) | 6.49 × 10^-9^ | 0.1090 (↑) | Included |
| rs429358 (C) | Dominant | -2.44 (-3.27 – -1.61) | 1.33 × 10^-8^ | 0.1070 (↓) | Excluded |
| rs7023003 (G) | Recessive | -2.61 (-3.46 – -1.75) | 3.29 × 10^-9^ | 0.1100 (↑) | Included |

MET-min/week: metabolic equivalent task minutes per week; 95%CI: 95% confidence interval
