# Supplementary material for: Genetic Background of Acute Heart Rate Response to Exercise
Source: Int J Mol Sci. 2024 Mar 13;25(6):3238. doi: 10.3390/ijms25063238 (PMC10970476; doi:10.3390/ijms25063238)
Supplement: Supplementary file 1 [file ijms-25-03238-s001.zip › ijms-2901201-supplementary/Supplementary Table S3.docx]

**Supplementary Table S3.** Trend analysis of heart rate related to cardiovascular fitness in relation to groups based on optimized polygenic score (oPGS) values.

|  | oPGS (1 – 5)  (n = 140) | oPGS (6 – 9)  (n = 357) | oPGS (10 – 14)  (n = 123) | *p* for trend |
| --- | --- | --- | --- | --- |
|  | Average (95%CI) | | |  |
| HR_rest_ | 78.15 (76.55 – 79.15) | 77.33 (76.27 – 78.39) | 76.97 (74.97 – 78.96) | 0.449 |
| HR_exerc_ | 123.94 (118.69 – 129.18) | 108.71 (106.18 – 111.23) | 102.72 (99.39 – 106.05) | <0.001** |
| HR_5min_ | 100.60 (97.31 – 103.89) | 92.11 (90.49 – 93.74) | 87.73 (85.50 – 89.96) | <0.001** |
| HR_10min_ | 85.71 (83.62 – 87.80) | 81.41 (80.21 – 82.62) | 79.74 (77.84 – 81.63) | <0.001** |
| ΔHR | 45.79 (40.45 – 51.12) | 31.38 (29.02 – 33.73) | 25.75 (23.14 – 28.60) | <0.001** |
| ΔHR_5min_ | 22.45 (19.19 – 25.71) | 14.78 (13.30 – 16.27) | 10.76 88.97 – 12.56) | <0.001** |
| ΔHR_10min_ | 7.56 (5.83 – 9.28) | 4.08 (3.17 – 5.00) | 2.77 (1.44 – 4.10) | 0.003** |

HR_rest_: resting heart rate; HR_exerc_: heart rate immediately after completing the physical exercise; HR_5min_: heart rate 5 minutes after the physical exercise; HR_10min_: heart rate 10 minutes after the physical exercise; ΔHR: delta heart rate defined as the difference between the heart rate immediately after completing the physical exercise and the resting heart rate; ΔHR_5min_: defined as the difference between the heart rate 5 minutes after physical exercise and the resting heart rate; ΔHR_10min_: defined as the difference between the heart rate 10 minutes after physical exercise and the resting heart rate. 95%CI: 95% confidence interval; **: significant *p*-value (<0.00625) after Bonferroni correction
