# Supplementary material for: Genetic Background of Acute Heart Rate Response to Exercise
Source: Int J Mol Sci. 2024 Mar 13;25(6):3238. doi: 10.3390/ijms25063238 (PMC10970476; doi:10.3390/ijms25063238)
Supplement: Supplementary file 1 [file ijms-25-03238-s001.zip › ijms-2901201-supplementary/Supplementary Table S4.docx]

**Supplementary Table S4.** Trend analysis of the average MET-min/week change in total, categories, and domains of physical activity compared to groups based on the optimized polygenic score (oPGS).

|  | oPGS (1 – 5) | oPGS (6 – 9) | oPGS (10 – 14) | *p* for trend |
| --- | --- | --- | --- | --- |
|  | Average MET-min/week (95%CI) | | |  |
| Total physical activity | 9,294  (8,104 – 10,484) | 10,690  (9,867 – 11,512) | 11,644  (10,172– 13,115) | 0.022* |
| By intensity categories | Average MET-min/week (95%CI) | | | *p* for trend |
| Vigorous | 2,301  (1,625 – 2,978) | 3,207  (2,644 – 3,771) | 3,540  (2,621 – 4,458) | 0.003** |
| Moderate | 5,338  (4,590 – 6,086) | 5,532  (5,058 – 6,005) | 6,020  (5,094 – 6,946) | 0.370 |
| Light | 1,655  (1,321 – 1,989) | 1,951  (1,712 – 2,189) | 2,084  (1,703 – 2,465) | 0.031* |
| By domains | Average MET-min/week (95%CI) | | | *p* for trend |
| Work | 4,195  (3,292 – 5,098) | 5,125  (4,469 – 5,782) | 5,055  (3,994 – 6,117) | 0.097 |
| Transport | 1,370  (1,075 – 1,666) | 1,466  (1,270 – 1,662) | 1,726  (1,312 – 2,140) | 0.888 |
| Domestic work and gardening | 2,809  (2,333 – 3,284) | 2,932  (2,639 – 3,226) | 3,206  (2,628 – 3,785) | 0.502 |
| Leisure-time | 920  (659 – 1,181) | 1,166  (982 – 1,349) | 1,656  (1,261– 2,050) | <0.001** |

MET-min/week: metabolic equivalent task minutes per week; 95%CI: 95% confidence interval; *: *p* <0.05; **: significant *p*-value (<0.00625) after Bonferroni correction).
