# Supplementary material for: Genetic Background of Acute Heart Rate Response to Exercise
Source: Int J Mol Sci. 2024 Mar 13;25(6):3238. doi: 10.3390/ijms25063238 (PMC10970476; doi:10.3390/ijms25063238)
Supplement: Supplementary file 1 [file ijms-25-03238-s001.zip › ijms-2901201-supplementary/Supplementary Table S5.docx]

**Supplementary Table S5.** The trend analysis results in the distribution of the composition of total physical activity by intensity category and domain for the subgroups based on the oPGS.

|  | oPGS (1 – 5) | oPGS (6 – 9) | oPGS (10 – 14) | *p* for trend |
| --- | --- | --- | --- | --- |
| By intensity categories | Average in % (95%CI) | | |  |
| Vigorous | 17.67  (13.76 – 21.58) | 21.49  (18.92 – 24.07) | 23.85  (19.44 – 28.27) | 0.006** |
| Moderate | 60.62  (56.45 – 64.80) | 57.00  (54.41 – 59.60) | 52.93  (48.29 – 57.57) | 0.014* |
| Light | 1.28  (0.53 – 2.03) | 0.77  (0.34 – 1.21) | 0.73  (0.41 – 1.06) | 0.102 |
| By domains | Average in % (95%CI) | | | *p* for trend |
| Work | 30.52  (25.16 – 35.89) | 34.96  (31.64 – 38.28) | 34.58  (29.01 – 40.14) | 0.186 |
| Transport | 20.83  (17.29 – 24.37) | 17.18  (15.32 – 19.05) | 16.37  (12.72 – 20.02) | 0.014* |
| Domestic work and gardening | 38.54  (34.27 – 42.82) | 35.97  (33.33 – 38.61) | 33.85  (29.03 – 38.68) | 0.060 |
| Leisure-time | 10.10  (8.57 – 12.64) | 11.89  (10.25 – 13.25) | 15.20  (12.20 – 18.20) | <0.001** |

95%CI: 95% confidence interval; *: *p* <0.05; **: significant *p*-value (<0.00625) after Bonferroni correction.
